# Influence of Sociodemographic, Health-Related, and Behavioral Factors on Food Guidelines Compliance in Older Adults: A Hierarchical Approach from the Chilean National Health Survey 2016–17 Data

**DOI:** 10.3390/geriatrics7020047

**Published:** 2022-04-08

**Authors:** Leticia de Albuquerque-Araújo, Daiana Quintiliano-Scarpelli, Dominique Masferrer Riquelme, Jair Licio Ferreira Santos

**Affiliations:** 1 Departamento de Salud Pública, Facultad de Ciencias de la Salud, Universidad de Talca, Talca 3460000, Chile; leticiadeaa@gmail.com; 2 Carrera de Nutrición y Dietética, Facultad de Medicina Clínica Alemana, Universidad del Desarrollo, Santiago 7610658, Chile; d.masferrer@udd.cl; 3 Escuela de Salud Pública, Facultad de Medicina, Universidad de Chile, Santiago 8380453, Chile; 4 Departamento de Medicina Social, Faculdade de Medicina, Universidade de São Paulo, Ribeirão Preto 14049-900, Brazil; jalifesa@usp.br

**Keywords:** diet, food guidelines, hierarchical models, living alone, lifestyle factors, elderly

## Abstract

Dietary habits are determinants in the development of a range of conditions and age-related diseases. We explored the associations of sociodemographic, health-related indicators, and health behavioral factors on dietary guideline compliance in elderly Chileans. We used a cross-sectional design using the publicly available database from the last Chilean National Health Survey (2016–17). The sample of 1831 older adults (≥60 y) from a national representative sample. The dependent variable was compliance with Food Guidelines (FG) (daily consumption of water, dairy, and fruits and vegetables; and weekly consumption of legumes and fish). The independent variables included sociodemographic, health-related, and behavioral factors. Over half (51.8%) of the sample was female and 85.7% belonged to the 60–79 age group. Satisfactory compliance to FG was observed in 3.9% of the sample. In the adjusted analysis, for those between 60 and 79 y, marital status was the only significant variable associated with FG noncompliance (PR: 1.34; 95%CI: 1.04–1.71). For those over 80 y, income of >2 minimum wages (PR: 0.10; 95%CI: 0.02–0.61), living alone (PR: 1.72; 95%CI: 1.20–2.47), and self-reported cardiovascular disease (PR: 0.63; 95%CI: 0.43–0.93) were associated with FG noncompliance. We observed low FG compliance among elderly Chilean adults, especially in the oldest group. Factors associated with the FG compliance was different between age groups.

## 1. Introduction

Population aging is a global phenomenon that poses different challenges depending on its magnitude, speed of occurrence, and the socioeconomic contexts in which it takes place [1]. Indeed, there are significant gaps related to life and healthy life expectancy across and within countries, differences of up to thirty-seven years for life expectancy at birth [1,2]. Chile’s demographic and epidemiological transitions are among the most advanced in Latin America. In 2020, the average life expectancy at birth was 80.3 years (both sexes) and 16.2% of the population was 60 years or older [3]. Furthermore, Chile is among the countries with the fastest growth of life expectancy at birth compared to European and North American countries, and the group that has experienced the highest growth rate is those >80 years, having more than tripled the life expectancy since 2015 [4].

Although increasing longevity is often assumed to be accompanied by an extended period of good health, little evidence supports the fact that older people are experiencing better health compared with their parents at the same age [3]. This has led to an increase in related research about epidemiological and social aspects of ageing, health and functional changes experienced with ageing, the impact of physical activity, the assessment of the nutritional status of older persons, and the development of nutritional guidelines for healthy ageing [5].

Most current perspectives have defined healthy ageing using a multidimensional approach connecting genetic, environmental, and behavioral factors, as well as socioeconomic and spatial determinants [1,6,7]. In this sense, healthy dietary habits are a fundamental aspect of healthy aging, considering that dietary changes seem to affect risk factor disease levels throughout life [8] and are significantly associated with improvement in at least one domain of quality of life in older adults [9]. However, ageing is associated with physiological, cognitive, social, and lifestyle changes that influence dietary intake and nutritional status [10,11].

Recent research has reported a moderate adherence to healthy dietary patterns among older adults [10,12,13], which has been associated with three broad domains: (i) changes associated with ageing, (ii) psychosocial aspects, and (iii) personal resources [14]. According to Host et al. [14], physiological changes associated with ageing have a significant impact on food choice. In particular, poor dentition, taste or chemosensory change, loss of appetite, illness or medical conditions, and mobility/functional limitations were identified as serving to shape decisions regarding foods consumed.

With respect to psychosocial aspects, six major domains are linked to food choice: life course, loneliness (and/or living arrangement), lack of motivation and/or energy, personal interest in health and/or nutrition, self-perception of health status, and desire for independence. Regarding personal resources, income/food costs, access to quality produce, transportation issues, knowledge and/or skills in food preparation, access to support, and individual dietary resilience were distinguished as determinants of food choice [14,15,16]. However, there appears to be a considerable lack of research on identifying and understanding how those factors interact with each other and influence food choices in people over 80 years, who experience more frequently sensory impairments and multimorbidity.

In Chile, nationally representative diet studies among the elderly population have not been conducted, and details of diet quality and access to healthy food are practically unknown in this group. In the National Health and Dietary Surveys [17,18], some nutritional data are available for this age-group but summaries often combine age groups (e.g., >60 y or >65 y), which is insufficient. As mentioned, the nutritional needs and functional capacities differ by age, and the needs of those >80 y are different from the needs of other elderly populations. Considering this framework, the purpose of our study was to explore the associations of socio-demographic, health-related indicators, and health behavioral factors on dietary guideline compliance in elderly Chileans considering a hierarchical approach.

## 2. Materials and Methods

### 2.1. Study Design and Sample

We analyzed data from the Chilean National Health Survey (NHS) conducted between August 2016 and March 2017. The NSH was cross-sectional and used a multistage stratified random sampling strategy, resulting in a final sample of 6233 persons ≥ 15 years old, with national, regional, and urban/rural representativeness with a 2.6% absolute sampling error. The NHS included the measurement of approximately 60 health problems or prioritized diseases and the assessment of risk and protective health factors such as physical activity level, food intake, and nutritional status. In addition, the NHS study design considered an expansion factor that allowed the representation of the entire (n = 14,518,969) Chilean population. Regarding the elderly population, the NHS dataset included 1831 older adults (≥60 y), representing 3,066,214 older Chilean adults, all of whom were included in this study. Data collection was carried out by face-to-face household interviews by standardized personnel. The overall response rate was 67% [18]. More detailed information about the survey has been described previously [19]. NHS protocols were approved by the Ethics Committee of the Pontificia Universidad Católica de Chile and all participants signed informed consent. This database is public and was fully anonymized by the Chilean Ministry of Health. 

### 2.2. Variables 

#### 2.2.1. Dependent Variable

To assess the dietary habits of older adults, five questions based on the Food Guidelines for the Chilean Population were considered. The answers from each question were categorized according to the food intake recommendation [20].

1. Daily water intake: <6 glasses; ≥6 glasses.
2. Weekly frequency of legumes (beans, lentils, peas, chickpeas) consumption: <2 times; ≥2 times.
3. Daily frequency of non-fat dairy intake: <3 servings; ≥3 servings.
4. Weekly frequency of fish and seafood: <2 times; ≥2 times.
5. Daily portions of fruits and vegetables: <5 portions; ≥5 portions.

Based on the Chilean National Food Consumption Survey (ENCA) [17], a global index of food intake compliance was created and grouped the subjects who meet the recommendations according to Chilean food guidelines. For this purpose, “satisfactory compliance” was considered if a compliance of at least three of the five guidelines was reported; a compliance with 1 or 2 guidelines was considered “partial compliance”; and “noncompliance” was considered when none of the 5 guidelines was reported. For this study, the noncompliance category was considered the main outcome.

#### 2.2.2. Independent Variables

**Figure 1:**
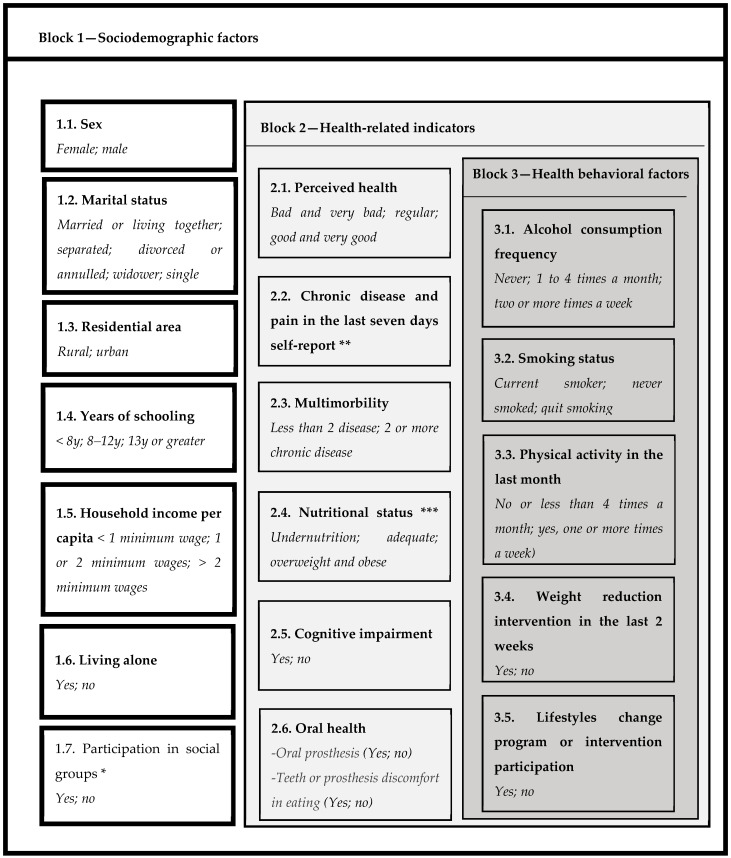
Hierarchical blocks and categories of independent variables. * Social groups: sports, volunteer, or hobby groups, senior citizen clubs, neighborhood or resident associations, study or cultural groups, religious organizations, or church participation. ** The self-report of chronic disease includes cardiovascular (hypertension, heart attack, stroke, and high cholesterol); eye disorders, such as cataracts and glaucoma; and osteoarticular diseases (arthritis, osteoarthritis, and osteoporosis). The pain self-report includes muscle, bone, or joint pain. *** The nutritional status categories were defined as undernutrition (BMI < 23 kg/m^2^), adequate (BMI 23.0–27.9 kg/m^2^), overweight (BMI 28.0–29.9 kg/m^2^), and obese (BMI ≥ 30 kg/m^2^).

The selected independent variables were those that have been shown to influence the diet of older adults [8,9,10,11,12,13,14,15,16]. Considering the conceptual framework, variables were grouped in three hierarchical blocks: sociodemographic, health-related, and health behavior factors (Figure 1). The first block included a set of sociodemographic variables such as years of schooling, income (based on current minimum wage in the period), living company, and social participation. A detailed description of all variables is provided in Figure 1.

The second block considered health-related factors such as perceived health, self-reported chronic diseases (osteoarticular, cardiovascular, and eye disorders), nutritional status assessment by Body Mass Index (BMI) according to the Pan American Health Organization (PAHO) cut-off points [21], oral health and prosthesis use considering impact on food intake, muscular and joint pain, and cognitive status (Figure 1). Cognitive status was assessed with a shortened version of the Mini-Mental State Examination (MMSE), which has a maximum score of 19 and a cut-off point of 13 with a combination of the Pfeffer Functional Activities Questionnaire (PFAQ], according to Quiroga et al. [22]. The criteria used to determine if participants had cognitive impairment were MMSE< 13 and PFAQ ≥ 6. When participants obtained a score of 12 or less on the MMSE, a relative or a person who lived in the household answered the PFAQ.

The third block included selected variables related to current lifestyle (alcohol consumption, smoking status, and physical activity level) and previous nutritional interventions that may interfere with food guidelines compliance, such as currently being in a weight loss program and previous participation in a lifestyle change program. The last one refers to intervention programs conducted in the primary health care centers recommended by Chilean public policy when an individual has diabetes, hypertension, or dyslipidemia.

### 2.3. Data Analysis

All performed analyses considered the survey sampling design. Descriptive data from categorical variables were expressed in relative frequencies (%). Multivariate robust Poisson regression was used to assess the association between food guideline compliance and the sociodemographic, health-related indicators, and behavioral factors, stratified by age group (60–79 years; ≥80 years). Variables included in the multivariate model were those with *p* < 0.20 in the bivariate model. The modeling procedure was stepwise forward. The variables from each block were inserted one by one; these remained as adjustment factors for the hierarchically lower blocks. Selected variables were maintained in the model even though their statistical significance was not preserved with the inclusion of lower hierarchical blocks. Associations between the factor and food guideline compliance after adjustment for the potential factors of the same block and the higher hierarchical indicators blocks were interpreted from the final model. The Akaike information criteria (AIC) were used to compare multiple models and determine which one was best. The lowest possible AIC score indicated the best balance of model fit [23]. Alpha was set at *p* < 0.05. Statistical analysis was carried out using STATA 14.1.

## 3. Results

In this study, 1826 subjects met the inclusion criteria, 51.8% were female, and 85.7% belonged to the 60–79 age group. In Table 1, the description of sociodemographic and health-related variables by age is presented. Significant differences between age groups were observed, with a higher prevalence of widowhood, low education level, bad and very bad perceived health, eye diseases, multimorbidity, cognitive impairment, and use of oral prosthesis in those ≥80 y (*p* < 0.05). Considering nutritional status, excess weight was present in 61.0% of participants under 80 y, and underweight in one sixth of the oldest group (*p* = 0.005).

The description of health behavioral factors showed a higher consumption of alcohol and smoking habits in the youngest age group. Physical activity practice at least once a week in the last month was low in the whole sample, but especially in those over 80 y (*p* < 0.05). Most subjects had participated in lifestyle change programs or treatments, and one quarter was trying to lose weight, especially in the group <80 y (Table 1).

Considering diet, satisfactory compliance to food guidelines was observed in only 3.9% of the sample. The legumes recommendation was the guideline most frequently accomplished, followed by water consumption. Dairy and fish consumption presented a low prevalence: 5.3% and 9.1%, respectively. A significant difference between age groups was observed for fruit and vegetable consumption, being lower in those over 80 y (Table 2).

Table 3 presents the hierarchical analysis (crude and adjusted) for elderly subjects between 60 and 79 y. In the crude analysis, among the sociodemographic factors, marital status (widower) and years of study were associated with noncompliance with dietary guidelines (*p* < 0.05). In the adjusted analysis, sex, marital status, and years of study were included, but only marital status remained significantly associated (being a widower versus being married or living together) (*p* < 0.05). For the block of health-related factors, the self-perception of health and report of osteoarticular disease were associated with noncompliance to dietary guidelines. Perceived health, report of osteoarticular disease, and the BMI were associated in the crude analysis, although none of them remained significantly in the adjusted hierarchical analysis. No variable from the health behaviors block was significantly associated with noncompliance in this age group.

For the group aged 80 years and over (Table 4), the sociodemographic block variables associated with noncompliance with dietary guidelines in the crude analysis were years of schooling, per capita household income, and living alone (*p* < 0.05). These variables were included in the adjusted analysis, but only income and living alone remained statistically significant. From the block of health-related factors, self-reported cardiovascular and ocular diseases and BMI were associated with noncompliance. These variables and the report of osteoarticular diseases were included in the adjusted hierarchical analysis, with cardiovascular disease remaining significant. Finally, in the block of health behavior factors, only smoking was associated and included in the adjusted analysis, which did not remain significant in the final model.

## 4. Discussion

The findings of this study suggest a high prevalence of noncompliance with dietary guidelines among elderly Chileans, highlighting a satisfactory compliance in less than 5% in both age groups. Fish and dairy products were the guidelines least frequently accomplished. The factors related to noncompliance to dietary guidelines were different between age-groups, finding a greater number of sociodemographic variables among adults 80 years and over.

Food-based dietary guidelines aim to promote good nutrition through easy and culturally adapted recommendations. In Chile, according to the Chilean National Dietary Survey (ENCA) [17], approximately 15.0% (95%CI 11.3–18.7) of those ≥65 y had good diet quality. This proportion is higher when compared to younger age group; however, several key micronutrients are considered deficient (calcium, vitamin A and B_12_, zinc) or excessive (e.g., sodium) [17].

Candia et al. [24] conducted a study among 458 elderly subjects (≥60 years from Santiago) to determine diet quality and observed that men consumed a higher number of unhealthy foods compared to women (*p* = 0.01) (sugary beverages, fried and junk food, sweet snacks, etc.), though both sexes showed similar healthy eating habits [24,25]. In age comparisons, those ≥80 years of age consumed fewer unhealthy foods (*p* = 0.01) compared to those <80, and obese subjects compared to nonobese had better eating habits.

International evidence agrees with this tendency; usually, older women compared to older men, and those >80y and with more chronic diseases present more adequate diets [12,26]. The present study did not find differences between sex, BMI, and age with respect to satisfactory compliance of the guidelines, only in the consumption of fruits and vegetables that was higher in the age group 60–79 years.

In our population, the lowest proportion of compliance was related to dairy and fish intake. The results showed a lower prevalence than that reported in ENCA data (dairy intake of 5.3% versus 19.4% and fish intake of 9.1% versus 13.1%) [17]. A low consumption of fish is prevalent in this population, even in countries with a Mediterranean diet [27]. In a study conducted among elderly Dutch persons, Dijkstra et al. [28] showed that lower-socioeconomic groups met the guidelines less often and perceived more barriers to adhere to the fruit, vegetable, and fish intake guidelines. The most frequently perceived barriers to meet guidelines were the high price of fruit and fish and a poor appetite for vegetables.

In relation to dairy consumption, the low consumption in our population may be due to previous habits, considering that younger Chilean adults also have a low intake of dairy [17,29] and could be related to the digestibility of these products at this age. Adequate dairy intake is related to several benefits specific to this age group [30]. The addition of nutrient-rich dairy proteins may improve physical performance and attenuate loss of muscle strength, thereby helping to prevent sarcopenia in the elderly population and reduce the risk of hypertension [31], mortality, and major cardiovascular disease events [32]. However, this depends on the type of dairy product and the quantity ingested. A systematic review [33] showed that the available evidence on benefits of dairy consumption among the elderly is related to some positive effects of dairy product intake on frailty, especially with high consumption of low-fat milk and yogurt.

Full diet compliance with intake guidelines is frequently related to health benefits, but few subjects achieve it. Hansen et al. [34] analyzed the compliance to Danish Dietary Guidelines in 55,021 elderly Danish subjects and found that a higher adherence was associated with a lower risk of cardiovascular disease. The inverse association was observed with full compliance and compliance with four or five of six guidelines. Compliance with the food-based dietary guidelines was low (0.04% complied fully with all six included guidelines and 7% complied with at least five guidelines). The lowest compliance was seen for the intake of fruits and vegetables and intake of whole grains, where the proportion of compliers was less than 10% [34].

Eating healthy in Chile has a higher cost than eating unhealthy [35], which justifies the finding that higher incomes decrease the possibility of showing FG noncompliance in the group aged 80 years and over, similar to the findings of other studies [36,37,38]. Income level is a recognized structural determinant of health, acting in various ways, including promoting more opportunities and knowledge to adopt better health behaviors, such as better eating habits [39]. According to Dunneram and Jeewon [40], lower income levels are associated with a greater risk of experiencing hunger and food insufficiency in elderly people and also limits the quantity and nutritional quality of foods purchased. Furthermore, the economic sensitivity of diet is considered particularly salient for older adults as they are more likely to have a low income due to a drop in income through retirement and the out-living of savings.

Thorpe et al. [41] studied Australian subjects and found that being male, having a higher level of education, not smoking, and meeting physical activity recommendations were predictors of a favorable change in dietary patterns. Compliance to the overall Australian Dietary Guidelines remained low, with a mean (SD) score of 83.1 (14.1) and 90.6 (13.1) for men and women, respectively, out of a total achievable score of 130. The design of nutrition promotion initiatives for older adults needs to consider those of lower socioeconomic status, as having a lower level of education was a predictor of poorer dietary patterns.

An interesting finding of this study was the fact that living alone was associated with a higher prevalence of FG noncompliance in those aged 80 and over, but not in the younger group. Persons over 80 years in Chile had worse perceived health, a higher prevalence of eye diseases, multimorbidity, poor nutritional status, cognitive impairment, and use of dental prosthesis compared to older adults aged 60 to 79 years. These factors combined with a context of living alone make it difficult to acquire fresh food, prepare meals, and even eat. Living alone is related to nutritional insufficiency in old age [14] and restricts the opportunities for commensality, the act of eating with others. Commensality, in turn, may promote opportunities for social integration, social support, and companionship to occur [42], which can encourage healthy food intake. In addition, eating is a social activity, and the absence of company can lead to loss of pleasure associated with eating and cooking. Older people who live alone report a lack of motivation to buy, prepare, and eat food. They often skip meals and replace nutritious meals with snacks, consume processed or convenience foods, and eat fewer and a lower variety of foods [15,16].

According to Payette and Shatenstein, widowhood affects the diet of those over 75 years of age more than those younger. However, in our study, this occurred only in the 60- to 79-year-old group. In our nationally representative study, being a widower increased the prevalence of noncompliance of dietary guidelines versus being married or living with a partner. Being a widower decreases "nutritional self-management," leading to changes in dietary behavior [43]. As eating habits is a behavioral health factor, the present study reinforces the relevance of social and contextual conditions in the adoption of healthy behaviors [40]. Social factors were more important in determining compliance with dietary guidelines than the other lifestyles factors in the current study.

Regarding multimorbidity, self-reported cardiovascular disease was associated with a lower prevalence of noncompliance of the guidelines in the age group 80 and over. Previous systematic reviews have shown that dietary interventions such as nutrition education and counselling are related with better dietary quality in elderly persons [44,45], strategies that are incorporated in the care of patients with cardiovascular diseases at the primary healthcare level in Chile. This can be attributed to reverse causality, which can be found in cross-sectional studies, such that when people find out about their illness, they could improve their eating habits [46].

This study should be interpreted considering the following limitations. First, the cross-sectional design did not allow causality to be demonstrated and, in the case of some of the independent variables, might be presented as reverse causality. Second, the use of self-reported health variables might not adequately reflect the clinical and behavioral reality of the subjects but had a good correlation with health diagnosis. Another limitation comes from the fact that the dietary assessment from the NHS did not allow us to affirm that these 6 food groups represent the total compliance of all Chilean food guidelines, mainly for not including questions regarding foods to avoid. The main strengths were related to the national representative sample used and the hierarchical analysis carried out. In addition, the dietetic approach of the study applied to the growing elderly population was notable.

## 5. Conclusions

This research revealed the influence that the social context has on the low compliance to food guidelines among elderly Chileans, especially in the oldest group. The factors associated with food guidelines compliance were different between age groups. In particular, living alone and lower income levels were associated with a greater risk of experiencing unsatisfactory healthy food intake among those ≥80 y, while for those 60 to 79 years, being widowed was the main related factor. Thus, when studying factors that influence the diet of older adults, it may be important to look at age groups separately.

Regarding health-related indicators, self-reported cardiovascular disease was associated with a lower prevalence of noncompliance in the age group 80 and over, probably due to a greater exposure to dietary interventions such as nutrition education and counselling, strategies incorporated in the care of patients with cardiovascular diseases at the primary healthcare level in Chile. Unexpectedly, no behavioral health factor was significantly associated with dietary guidelines’ compliance in this study. Thus, these results provide evidence regarding the importance of the social environment for food intake among older adults.

Considering the essential contribution of food guideline compliance to healthy ageing, further studies are needed to enhance the current knowledge related to the effectiveness of social and community interventions designed to influence the dietary choices of older populations.

## Figures and Tables

**Table 1:** Description of sociodemographic and health-related indicators of the sample by age group.

Variables	Overall	60–79 y	≥80 y	*p*-Value ^‡^
**Population represented**	3,066,214	2,627,745	438,469	
**Sociodemographic factors**				
Female sex	51.8	51.4	54.2	0.652
Marital status				<0.001
Married or living together	59.9	63.6	37.3	
Separated, divorced, or annulled	10.8	11.5	6.4	
Widower	19.4	14.8	47.6	
Single	9.9	10.2	8.7	
Residential area				0.132
Urban	86.0	86.7	82.0	
Rural	14.0	13.3	18.0	
Years of schooling				<0.001
<8 y	46.1	41.9	71.9	
8–12 y	37.5	40.1	21.7	
≥13 y	16.4	18.0	6.4	
Household income per capita				0.774
<1 minimum wage	80.8	80.2	84.4	
1 to 2 minimum wages	14.1	14.4	12.1	
>2 minimum wages	5.1	5.4	3.5	
Living alone	19.2	18.2	24.7	0.106
Participation in social groups	35.9	35.8	36.3	0.929
Someone’s caretaker	21.6	24.0	6.5	<0.001
**Health-related indicators**				
Perceived health				<0.001
Bad and very bad	14.3	11.9	29.3	
Regular	42.4	43.1	38.2	
Good and very good	43.3	45.1	32.5	
Chronic disease self-report				
Cardiovascular diseases	73.0	71.6	81.4	0.082
Eye disorders and diseases	23.7	20.4	44.0	<0.001
Osteoarticular diseases	36.3	35.0	43.6	0.134
Multimorbidity (≥2 NCD) *	63.2	60.0	83.1	<0.001
Nutritional status				
Underweight	9.2	8.0	16.8	0.005
Adequate weight	32.3	31.0	40.2	
Overweight	20.5	21.1	16.5	
Obesity	38.0	39.9	26.5	
Cognitive impairment	4.2	2.3	15.6	<0.001
Oral prosthesis	60.2	58.3	71.3	0.031
Teeth or prosthesis discomfort in eating	25.2	24.3	30.7	0.233
Muscles/bones/joint pain in the last 7 days	50.0	49.8	51.2	0.818

Data are expressed as proportions. ^‡^ Pearson’s χ^2^ test. NCD: noncommunicable diseases; BMI: body mass index. * Self-report of hypertension, diabetes, high cholesterol, heart attack, stroke, glaucoma, cancers, osteoarticular diseases, kidney failure, lung disease, and cataracts.

**Table 2:** Description of health behavioral and dietary factors of the sample by age group.

Variables	Overall	60–79 y	≥80 y	*p*-Value ^‡^
**Health behavioral factors**				
Alcohol consumption frequency				
Never	41.5	39.3	55.2	0.018
1 to 4 times a month	48.0	49.7	37.5	
Two or more times a week	10.5	11.0	7.2	
Smoking status				
Never smoked	46.1	43.6	60.8	0.035
Quit smoking	37.9	38.9	32.0	
Current smoker	16.0	17.5	7.2	
Physical activity in the last month				
No	89.3	88.3	95.3	0.016
Yes	10.7	11.7	4.7	
Weight reduction intervention in the last 2 weeks	26.6	29.6	8.9	<0.001
Lifestyles change program or intervention participation	64.3	63.4	69.7	0.362
**Food Guidelines**				
5 servings of fruits and/or vegetables a day	19.3	20.4	12.9	0.045
Fish and seafood at least twice a week	9.1	9.5	6.6	0.343
Legumes at least twice a week	30.6	30.2	33.3	0.585
At least 6 glasses of water a day	20.2	20.9	16.0	0.225
3 servings of dairy a day	5.3	5.0	7.4	0.287
**Food Guidelines Compliance**				
Noncompliance	41.1	40.9	42.7	0.627
Partial compliance	55.0	55.0	54.8	
Satisfactory compliance	3.9	4.1	2.5	

Data are expressed as proportions. ^‡^ Pearson’s χ^2^ test.

**Table 3:** Crude and hierarchically adjusted associated factors to food guideline compliance in elderly Chileans between 60 and 79 y.

Variables	Noncompliance	Crude Model			Adjusted Model		
	%	PR	95%CI	*p*-Value ^1^	PR	95%CI	*p*-Value ^1^
**Block 1—Sociodemographic factors**							
Female sex	43.9	1.16	0.94–1.44	0.162	1.16	0.94–1.43	0.180
Marital status							
Married or living together	37.2	1.00	-		1.00	-	
Separated, divorced, or annulled	46.4	1.25	0.91–1.71	0.168	1.18	0.86–1.60	0.301
Widower	51.6	1.39	1.08–1.78	0.010	1.34	1.04–1.71	0.021
Single	41.6	1.12	0.78–1.60	0.547	1.06	0.73–1.53	0.764
Rural residence	39.8	0.97	0.78–1.21	0.783			
Years of schooling							
<8 y	42.7	1.00	-		1.00	-	
8–12 y	34.8	0.82	0.65–1.02	0.073	0.85	0.68–1.06	0.147
≥13 y	48.4	1.13	0.82–1.56	0.442	1.21	0.89–1.65	0.232
Household income per capita							
<1 minimum wage	41.8	1.00	-				
1 to 2 minimum wages	30.8	0.74	0.45–1.21	0.223			
>2 minimum wages	38.0	0.91	0.48–1.71	0.769			
Living alone	39.5	0.96	0.77–1.19	0.707			
Participation of social groups	43.2	1.11	0.89–1.37	0.353			
**Block 2—Health-related indicators**							
Perceived health							
Bad, very bad, and regular	45.0	1.00	-		1.00	-	
Good and very good	35.8	0.80	0.64–1.00	0.046	0.84	0.68–1.04	0.117
Chronic disease self-report							
With cardiovascular diseases	41.3	1.04	0.80–1.35	0.793			
With osteoarticular diseases	46.7	1.24	1.03–1.49	0.025	1.17	0.97–1.41	0.103
With eye disorders and diseases	43.4	1.08	0.85–1.37	0.537			
Nutritional status							
Adequate weight	40.6	1.00	-		1.00	-	
Underweight	33.9	0.84	0.56–1.25	0.386	0.82	0.56–1.22	0.335
Overweight	51.6	1.27	0.92–1.75	0.147	1.26	0.92–1.73	0.158
Obesity	36.8	0.91	0.71–1.17	0.448	0.87	0.258	0.258
With Cognitive impairment	50.6	1.25	0.73–2.13	0.423			
Uses oral prosthesis	38.8	0.88	0.73–1.08	0.222			
Teeth or prosthesis with discomfort in eating	37.3	0.89	0.70–1.13	0.321			
**Block 3—Health behavioral factors**							
Alcohol consumption frequency							
Never	40.6	1.00	-				
1 to 4 times a month	42.2	1.04	0.85–1.28	0.707			
Two or more times a week	35.7	0.88	0.53–1.47	0.626			
Smoking status							
Never smoked	42.7	1.00	-				
Quit smoking	39.0	0.91	0.72–1.16	0.464			
Current smoker	40.7	0.95	0.72–1.26	0.732			
Physical activity in the last month	33.7	0.81	0.52–1.26	0.341			
Weight reduction intervention in the last 2 weeks	37.4	1.09	0.87–1.36	0.477			
Lifestyles change program or intervention participation	42.1	0.88	0.68–1.16	0.365			

PR: prevalence ratio; CI: confidence interval. ^1^ Robust Poisson Regression. AIC Sociodemographic Block: 3,966,999.0. AIC Sociodemographic and Health-related indicators block: 4,011,884.0.

**Table 4:** Crude and hierarchically adjusted associated factors to food guideline compliance in elderly Chileans ≥80 y.

Variables	Noncompliance	Crude Model			Adjusted Model		
	%	PR	95%CI	*p*-Value ^1^	PR	95%CI	*p*-Value ^1^
**Block 1—Sociodemographic factors**							
Female sex	41.7	0.95	0.59–1.55	0.840			
Marital status							
Married or living together	39.6	1.00	-				
Separated, divorced, or annulled	23.1	0.59	0.19–1.81	0.350			
Widower	47.5	1.20	0.71–2.02	0.491			
Single	44.1	1.12	0.47–2.66	0.805			
Rural residence	51.7	1.27	0.84–1.93	0.262			
Years of schooling							
<8 y	46.2	1.00	-		1.00	-	
8–12 y	24.2	0.52	0.29–0.94	0.030	0.56	0.29–1.07	0.077
≥13 y	69.0	1.49	0.74–3.00	0.258	0.94	0.60–1.47	0.783
Household income per capita							
<1 minimum wage	39.6	1.00	-		1.00	*-*	
1 to 2 minimum wages	76.2	1.92	1.27–2.91	0.002	1.33	0.90–1.98	0.157
>2 minimum wages	2.4	0.06	0.01–0.49	0.005	0.10	0.02–0.61	0.013
Living alone	64.1	1.80	1.24–2.61	0.002	1.72	1.20–2.47	0.003
Participation of social groups	32.2	0.66	0.38–1.13	0.126	0.81	0.46–1.42	0.455
**Block 2—Health-related indicators**							
Perceived health							
Bad, very bad, and regular	42.1	1.00	-				
Good and very good	43.9	1.04	0.65–1.67	0.857			
Chronic disease self-report							
With cardiovascular diseases	36.8	0.54	0.37–0.80	0.002	0.63	0.43–0.93	0.019
With osteoarticular diseases	35.4	0.73	0.46–1.16	0.183	0.74	0.49–1.13	0.164
With eye disorders and diseases	53.3	1.55	1.00–2.40	0.049	1.53	1.00–2.35	0.048
Nutritional status							
Adequate weight	31.3	1.00	-		1.00	-	
Underweight	59.5	1.90	1.08–3.36	0.027	1.73	0.93–3.23	0.084
Overweight	62.2	1.99	1.10–3.58	0.022	1.29	0.76–2.17	0.339
Obesity	37.3	1.19	0.65–2.19	0.570	1.59	0.92–2.76	0.099
With Cognitive impairment	44.2	1.04	0.61–1.77	0.879			
Uses oral prosthesis	41.1	0.88	0.52–1.49	0.642			
Teeth or prosthesis with discomfort in eating	33.6	0.72	0.40–1.29	0.264			
**Block 3—Health behavioral factors**							
Alcohol consumption frequency							
Never	44.8	1.00	-				
1 to 4 times a month	41.4	0.92	0.54–1.56	0.763			
Two or more times a week	33.1	0.74	0.34–1.61	0.441			
Smoking status							
Never smoked	31.7	1.00	-		1.00	-	
Quit smoking	55.9	1.76	1.14–2.72	0.011	1.51	0.99–2.30	0.055
Current smoker	76.6	2.42	1.44–4.06	0.001	1.19	0.75–1.88	0.456
Physical activity in the last month	27.9	0.64	0.19–2.20	0.479			
Weight reduction intervention in the last 2 weeks	36.4	0.68	0.40–1.17	0.167			
Lifestyles change program or intervention participation	37.5	0.84	0.41–1.71	0.628			

PR: prevalence ratio; CI: confidence interval. ^1^ Robust Poisson Regression. AIC Sociodemographic Block: 512,447.5; AIC Sociodemographic and Health-related indicators block: 518,969.6; AIC Sociodemographic, Health-related indicators, and Behavior factors blocks: 522,763.2.

## Data Availability

The raw data supporting the conclusions of this article will be made available by the authors, without undue reservation.
